# Plasma-Polymer-Fluorocarbon Thin Film Coated Nanostructured-Polyethylene Terephthalate Surface with Highly Durable Superhydrophobic and Antireflective Properties

**DOI:** 10.3390/polym12051026

**Published:** 2020-05-01

**Authors:** Eunmi Cho, Mac Kim, Jin-Seong Park, Sang-Jin Lee

**Affiliations:** 1 Chemical Materials Solutions Center, Korea Research Institute of Chemical Technology, Daejeon 34114, Korea; jommy@krict.re.kr (E.C.); kimmac79@krict.re.kr (M.K.); 2 Department of Materials Science and Engineering, Hanyang University, Seoul 04763, Korea; jsparklime@hanyang.ac.kr

**Keywords:** oxygen plasma treatment, nanostructure, plasma-polymer-fluorocarbon, antireflective effect, superhydrophobic, durability

## Abstract

Herein, an antireflection and superhydrophobic film was obtained by uniformly forming nanostructures on the surface of polyethylene terephthalate (PET) substrate using oxygen plasma without a pattern mask and coating plasma-polymer-fluorocarbon (PPFC) on the nanostructured surface by mid-range frequency sputtering. PPFC/nanostructured-PET showed a reflectance of 4.2%, which is 56% lower than that of the PET film. Haze was also improved. Nanostructured-PET exhibited a superhydrophilic surface due to plasma deformation and a superhydrophobic surface could be realized by coating PPFC on the nanostructured surface. The PPFC coating prevented the aging of polymer film nanostructures and showed excellent durability in a high-temperature and high-humidity environment. It exhibited excellent flexibility to maintain the superhydrophobic surface, even at a mechanical bending radius of 1 mm, and could retain its properties even after repeated bending for 10,000 times.

## 1. Introduction

Plasma is a very useful tool in polymer science. It is applied in a wide variety of fields, such as surface treatment, polymerization, nanostructure formation, and polymer coating, and has been actively researched and developed by many researchers [1]. Plasma can impart various functions, such as self-cleaning [2,3], antireflection [4], adhesion [5], biocompatibility [6], and friction [7] to the polymer surface.

The formation of nanostructures on a polymer surface using plasma is a very interesting process. Polymers with nanostructured surfaces have various properties, such as superhydrophobicity, pattern formation, antireflection, and biocompatibility, and can be used in various fields such as in automobiles [8], waterproof textiles [9], printing parts [10,11], displays [12], solar cells [13], bio-sensing [14], and medical devices [15]. Nanostructures can be formed by plasma etching or by coating only open regions using a pattern mask [16,17]. On the contrary, research is being actively conducted to form nanowrinkles, nanopores, and nanoprotrusions by plasma etching and deformation on the entire surface of the polymer substrate without a mask [16,17,18]. The maskless patterning method has significant advantages, e.g., low cost, simple process, and large area. However, there are still some challenges, such as uniform pattern formation, reaction gas selection, process condition optimization, mechanism identification, and reliability. Of many plasma generation supplies, linear ion source (LIS) with a closed drift type provides stable ionic energy and can irradiate a uniform ion beam over a large area, so it is used for the purpose of film cleaning and improving adhesion of a coating layer in a large-area coating processes by mounting it on roll-to-roll coating equipment. However, despite these advantages, few studies have been reported on forming nanostructures using LIS as a patterning method without mask.

Nanoscale polymer thin film coating using plasma is an emerging research field, and chemical vapor deposition, atomic layer deposition, and sputtering are the typically used processes in this regard. Among these processes, sputtering using a polymer target possess several advantages, such as easy thickness control, large area, and excellent adhesion. However, there are several disadvantages, such as the need to use a radio frequency power source due to the insulating properties of the polymer material, low deposition rate, and difficulty in long-time coating due to target deformation, which need to be resolved. Our group research has been conducting research on plasma-polymer-fluorocarbon (PPFC) thin films with various properties by mid-range frequency (MF) sputtering these films with a composite polymer target containing a conductive carbon nanotube (CNT) in a fluorine polymer. The carbon–fluorine ratio in the PPFC thin films produced by sputtering can be easily controlled. Therefore, these films can realize various physical properties, such as high transmittance, water repellency, chemical stability, and antibacterial properties [19,20,21,22,23]. PPFC thin films have a low refractive index, i.e., 1.39, and an amorphous structure. Therefore, they can be widely used in optical applications.

In this study, the optical properties of nanostructured-polyethylene terephthalate (PET) and PPFC/nanostructured-PET films were investigated by uniformly forming nanostructures on the surface of the PET substrate using an oxygen plasma through a LIS and coating the PPFC thin film on the nanostructured surface by MF sputtering. In addition, by coating PPFC with low surface energy on the nanostructured-PET surface, a superhydrophobic surface could be realized, and the structural, environmental, and mechanical reliabilities of the superhydrophobic film were evaluated.

## 2. Materials and Methods

### 2.1. Preparation of Nanostructured-PET Films

The surface of PET (100 μm, HYNT) was treated with an oxygen plasma using a linear ion source (LIS) to form nanostructures on the PET surface. The LIS chamber was evacuated to a base pressure of 1.0 × 10^−6^ Torr by a mechanical pump and turbo molecular pump. The O_2_ gas flow rate was 60 sccm and working pressure was approximately 3.5 × 10^−5^ Torr. A direct current power supply (Forte I-302, EN Technologies Inc., Gunpo, Korea) was used, with discharge voltage and current as 780 V and 0.05 A, respectively. The PET films were exposed to oxygen plasma at intervals of 100 s from 0 to 400 s.

### 2.2. Preparation of PPFC/Nanostructured-PET Films

**Figure 1:**
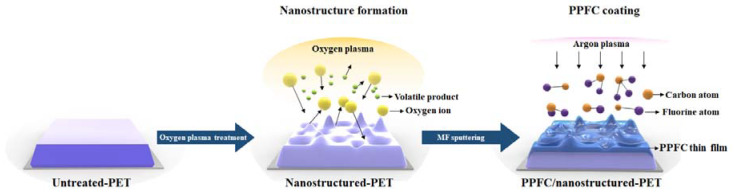
Schematic of the nanostructure formation and plasma-polymer-fluorocarbon (PPFC) thin film coating process.

The PPFC thin films were coated by MF sputtering using a CNT–polytetrafluoroethylene (PTFE) (5:95, w/w) composite target. The sputtering chamber was evacuated to a base pressure of 5.0 × 10^−6^ Torr by a mechanical pump and cryogenic pump. The CNT–PTFE composite target was sputtered at the working pressure of 2.0 × 10^−3^ Torr. Pure Ar gas was used as the sputtering gas, and its flow rate was controlled by a mass flow controller. The target-to-substrate distance was maintained at 240 mm. The PPFC thin films were deposited by sputtering using an MF power supply (PE II, Advanced Energy, Denver, CO, USA). The frequency and power of the MF power supply were 40 kHz and 300 W, respectively. The thicknesses of the PPFC thin films were tuned to 30, 60, and 100 nm by adjusting the coating time. The overall process of the preparation of PPFC/nanostructured-PET films is shown in Figure 1. 

### 2.3. Characterizations

The surface characteristics of the nanostructured-PET samples, fabricated by the oxygen plasma treatment, were analyzed by field-emission scanning electron microscopy (FE-SEM, MAIA3, TESCAN, Brno, Czech Republic) and atomic force microscopy (AFM, Nanoscope, Bruker, Billerica, MA, USA). The AFM was conducted in the tapping mode. The scan rate was 1 Hz, and the scan area was 3 μm × 3 μm. The chemical analysis of the nanostructured-PET samples was conducted using X-ray photoelectron spectroscopy (XPS, AXIS NOVA, Kratos, Manchester, UK). X-rays were generated by accelerating thermal electrons at a voltage/current of 15 keV/10 mA. The degree of vacuum in the XPS measurement was ~10^−9^ Torr, and the X-ray was monochromatic Al-Kα (1486.6 eV). The wetting characteristics of the sample surface were analyzed by measuring the water contact angle (WCA). The WCA was measured using a contact angle analyzer (Phoenix 300 touch, Surface Electro Optics, Suwon, Korea). The amount of the deionized water droplets used herein was 5.0 μL. The WCA was systematically analyzed using the obtained images. The optical transmittance and reflectance were measured in the wavelength range of 400 to 1200 nm using an optical spectrometer (U-4100, Hitachi, Tokyo, Japan). The reflectance was measured using a spectral reflectance attachment to allow light to enter the opening at a 5° slope. Transmission haze was measured using a haze meter (NDH-5000, Nippon Denshoku, Tokyo, Japan).

The durability of PPFC/nanostructured-PET films was determined by conducting an accelerated life test and a mechanical bending test. The accelerated life of the films was evaluated by observing the change in WCA over time for 15 days after placing the samples in a thermos–hygrostat (TH-TG-408, Jeio Tech, Daejeon, Korea) at 85 °C and relative humidity of 85%. Several samples were simultaneously placed in the thermos–hygrostat and taken out one per day to measure the WCA. The mechanical flexibility and cyclic fatigue characteristics for the inner and outer bending were tested using a bending test system (JIRBT-610, JUNIL TECH, Daegu, Korea). The mechanical flexibility of the samples was evaluated by measuring the bending radius. The WCA was measured after the bending test, which reduced the bending radius from 10 to 1 mm. The bending test was performed on both the inner and outer sides of the sample. Additionally, a cyclic fatigue test was conducted for 10,000 bending cycles at 100 mm/s with the bending radius fixed at 5 mm. For the same sample, the WCA was measured per 500 cycles, and the cycle was repeated.

## 3. Results and Discussion

### 3.1. Formation of the Nanostructured-Polymer Surface

**Figure 2:**
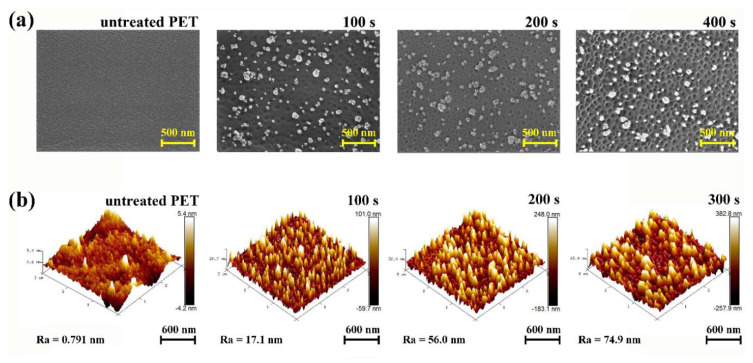
Morphological changes in nanostructured-polyethylene terephthalate (PET): (**a**) Field-emission scanning electron microscopy (FE-SEM) images of untreated-PET and nanostructured-PET (oxygen plasma treatment time from left 100, 200, and 400 s) and (**b**) Atomic force microscopy (AFM) 3D images of untreated-PET and nanostructured-PET (oxygen plasma treatment time from left 100, 200, and 300 s).

To form an effective nanostructure for antireflective, PET was treated with an oxygen plasma using a closed drift ion beam. The conditions for the oxygen plasma treatment were as follows: a power of 40 W and an oxygen gas flow rate of 60 sccm. Surface changes were observed at the plasma treatment times of 100, 200 and 300 s. The effects of power and oxygen flow rate are shown in the Supplementary Materials (Figure S1). Figure 2a shows the FE-SEM images of the surface of the polymer substrate obtained using different oxygen plasma treatment times. Although the untreated-PET surface is very clean and flat, the surfaces of the PET substrates treated with oxygen plasma have randomly distributed nanopores (dark) and nanoprotrusions (bright). The shapes and sizes of the nanopores and nanoprotrusions were relatively uniform. The nanopores and nanoprotrusions on the PET film are formed in physical and chemical etching reactions by various chemical species such as electrons, ions, and radicals present in the oxygen plasma. The nanopores are formed in a physical etching by oxygen ions and electron bombardment on the PET surface. On the other hand, the nanoprotrusions are formed in a chemical etching reaction by reactive plasma species (e.g., radicals, ions) that have penetrated not only the PET surface but also the bulk [1]. The reactive plasma species penetrating into the bulk decompose the ester pendant group of PET, which creates gas molecules. When the concentration of gas molecules in the bulk exceeds the solubility, gas bubbles are released to the surface, which form nanoprotrusions [10]. For PET films, nanowrinkles are formed at the ion energy of 200 eV, nanopores and nanoprotrusions are formed at 500 eV, and the aspect ratio of nanopores and nanoprotrusions increases to 800 eV [24,25]. In this study, since the oxygen plasma treatment was conducted at the ion energy of approximately 780 eV, the nanopores and nanoprotrusions were simultaneously formed. These nanopores and nanoprotrusions became more apparent with an increase in the plasma treatment time, and deterioration occurred on the surface of the PET substrate when the plasma treatment time was 500 s or more. Figure 2b shows the AFM images of the nanostructured-PET surface. The surface roughness (Ra) of untreated-PET is 0.79 nm, whereas the Ra values of the PET substrates treated with oxygen plasma for 100, 200, and 300 s have significantly increased to 17.1, 56.0, and 74.9 nm, respectively. Since the oxygen plasma treatment time increased, the energy transmitted by gas particles through collision increased, which resulted in nanopores with a depth ranging from ~60 nm to ~255 nm. The diameter of the nanopores gradually increased from ~150 nm to ~250 nm. The heights of the nanoprotrusions increased from 10 nm to 400 nm depending on the plasma treatment time, and the average diameter at the bottom of the nanoprotrusions was 250–300 nm. The AFM results of nanostructures for each plasma treatment time considered herein are presented in the Supplementary Materials (Table S1).

**Figure 3:**
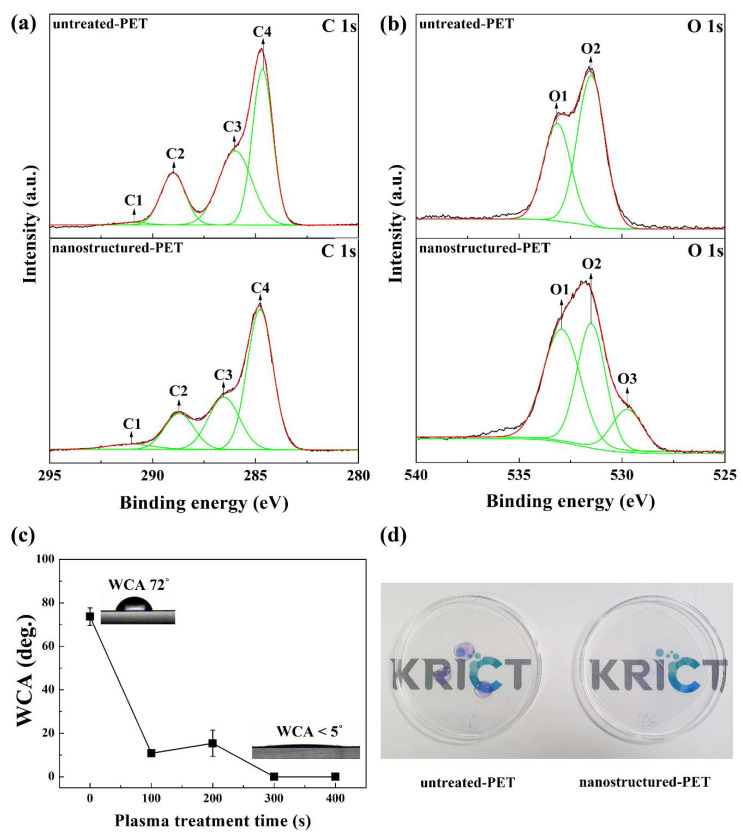
X-ray photoelectron spectroscopy (XPS) spectra and superhydrophilicity of nanostructured-PET: (**a**) C 1 s spectra, (**b**) O 1 s spectra of untreated-PET and nanostructured-PET, (**c**) changes in water contact angle (WCA) at different oxygen plasma treatment times, and (**d**) image showing the superhydrophilicity of nanostructured-PET.

Figure 3 shows the XPS spectra suggesting the chemical structure changes in the nanostructured-PET surface formed by the oxygen plasma treatment. Figure 3a shows the normalized C 1s core-level XPS spectra of the untreated-PET and nanostructured-PET films. Both spectra present four peaks assigned to shake-up satellite (C1 at 291.0 eV), O–C=O (C2 at 288.6 eV), C–O (C3 at 286.0 eV), and C–C (C4 at 284.6 eV). The position of C3 for nanostructured-PET shifted from 286.0 to 286.5 eV. This is because the oxygen ions in the plasma react with the chain of the ester group in PET to form a C=O bond on the film surface. A peak is formed at the same position as that of the C–O bond. Figure 3b shows the normalized O 1s core-level XPS spectra. The XPS spectra of untreated-PET exhibit two peaks assigned to the O=C (O1 at 533.1 eV) and O–C (O2 at 531 eV) bonds in the ester group. In the nanostructured-PET spectra, an additional peak (O3) is observed at 530 eV because hydrocarbons adsorb on the surface of PET upon its exposure to the atmosphere, which results in the formation of C–OH groups. As shown in Supplementary Materials (Table S2), the atomic concentration of oxygen on the PET surface increased after the oxygen plasma treatment, which increased the O/C ratio from 0.258 to 0.376. This is because the surface energy of nanostructured-PET increases due to the formation of oxygen-containing functional groups on the surface by oxygen plasma treatment. Therefore, the PET surface becomes hydrophilic. Figure 3c shows the changes in the WCA on the PET surface. Untreated-PET has a WCA of approximately 72°. However, the WCA rapidly decreases upon the oxygen plasma treatment, and the nanostructured-PET surface exhibits a superhydrophilicity of less than 5° when the oxygen plasma treatment time is 300 s or more. This is due to the formation of nanostructures and an increase in the number of oxygen-containing functional groups by plasma treatment. Therefore, it is possible to not only create uniform nanostructures on the PET surface through oxygen plasma treatment but also easily realize a superhydrophilic surface. Figure 3d shows water droplets placed on the nanostructured-PET and untreated-PET surfaces to show the superhydrophilicity of nanostructured-PET. On the superhydrophilic nanostructured-PET surface, the water droplets are in close contact with the surface and the shape of water droplets is not visible.

### 3.2. Antireflective Properties of PPFC/Nanostructured-PET Films

**Figure 4:**
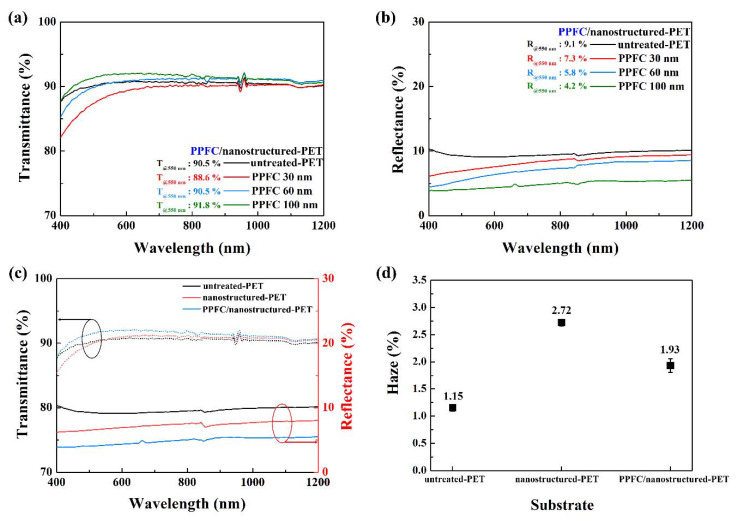
Optical characteristics of PPFC/nanostructured-PET. (**a**) Optical transmittance and (**b**) reflectance spectra of PPFC/nanostructured-PET as a function of PPFC coating layer thickness, (**c**) optical transmittance and reflectance spectra of nanostructured-PET and PPFC/nanostructured-PET, and (**d**) haze of untreated-PET, nanostructured-PET, and PPFC/nanostructured-PET.

The light incident on the microscale structure is partially absorbed and then reflected or scattered. However, when light enters a nanoscale structure with dimensions smaller than the wavelength of light, the surface interacts as if it has a gradient refractive index. The light is transmitted to reduce reflection [26,27]. The transmittance and reflectance of nanostructured-PET treated with oxygen plasma for 300 s were 90.5% and 6.7%, respectively, at a wavelength of 550 nm. Moreover, the transmittance was maintained and reflectance decreased when compared to those of untreated-PET (transmittance: 90.5% and reflectance: 9.1%). To increase the optical properties of the nanostructures, a PPFC thin film with a low refractive index (n ≈ 1.39) was coated on the nanostructured-PET surface. The transmittance and reflectance results, according to the PPFC thickness, are shown in Figure 4a,b, respectively. When the PPFC thickness was 0 (untreated-PET), 30, 60, and 100 nm, the transmittance was 90.5%, 88.6%, 90.5%, and 91.8% and the reflectance was 9.1%, 7.3%, 5.8%, and 4.2%, respectively, at a wavelength of 550 nm. When the PPFC thickness was 100 nm, an excellent improvement in the transmittance and reflectance was observed, and the reflectance was reduced to approximately 54% when compared to that of untreated-PET. The gradient refractive index was calculated using the Bruggeman effective refractive index equation [27].

(1) $$F_{2}\left(\frac{n_{\mathrm{eff}}{\;-\;n}_{2}}{n_{\mathrm{eff}}{\;-\;n}_{2}}\right)+F_{1}\left(\frac{n_{\mathrm{eff}}{\;-\;n}_{1}}{n_{\mathrm{eff}\;}{-\;n}_{1}}\right){+F}_{0}\left(\frac{n_{\mathrm{eff}}{\;-\;n}_{0}}{n_{\mathrm{eff}}{\;-\;n}_{0}}\right)=0$$

where n_eff_ is the effective refractive index, *n*_0_ is the refractive index of air (n_@550nm_ = 1.00), *n*_1_ is the refractive index of PET (n_@550nm_ = 1.70), *n*_2_ is the refractive index of PPFC (n_@550nm_ = 1.39), *F*_0_ is the areal fraction of air in each slice, *F*_1_ is the areal fraction of PET in each slice, and *F*_2_ is the areal fraction of PPFC in each slice.

At the top of the nanostructures, air occupies most of the area. Thus, *F*_0_ has a value close to 1, *F*_1_ has a value close to 0, and the area occupied by the nanostructures increases toward the bottom. Thus, *F*_0_ converges to 0 and *F*_1_ converges to 1. Essentially, the refractive index gradually increases from 1.0 to 1.7 from the top area to the bottom area. Therefore, as the cross-sectional area of the nanostructures is gradually widened and the refractive index is increased, the incident light can pass through the nanostructure without being reflected. Consequently, the reflection of the incident light on the nanostructures is prevented [28]. The previously mentioned results stating that the antireflective effect is maximized at the incident visible light wavelength of 200 nm diameter and 400 nm height support our results [29]. Therefore, by coating PPFC with a low refractive index on nanostructured-PET, excellent transmittance and antireflective characteristics were obtained. Figure 4c shows the transmittance and reflectance of untreated-PET, nanostructured-PET, and PPFC/nanostructured-PET samples. By coating 100-nm-thick PPFC on nanostructured-PET, the transmittance was significantly increased. The reflectance was significantly reduced only by forming nanostructures on the PET surface. Additionally, upon coating 100-nm-thick PPFC on nanostructured-PET, the antireflective effect became significant, which indicates a minimum reflectance of 4.2%. Figure 4d shows a graph indicating the haze of each sample. The haze of nanostructured-PET is 2.72%, which is more than twice as compared to 1.15% of untreated-PET. This is because light is scattered by relatively large nanoprotrusions (diameter: 350 nm, height: 400 nm or more) generated in nanostructured-PET. Due to the coating of the PPFC thin film on nanostructured-PET, the haze value decreases from 2.72% to 1.93%. The low refractive index of the PPFC thin film increases the transmittance and, thus, reduces the scattering of light. In conclusion, by forming uniform nanostructures on the PET surface using oxygen plasma and then coating a PPFC thin film on it, it is possible to realize an excellent optical film.

### 3.3. Superhydrophobic Surface and Durable Properties of PPFC/Nanostructured-PET Films

**Figure 5:**
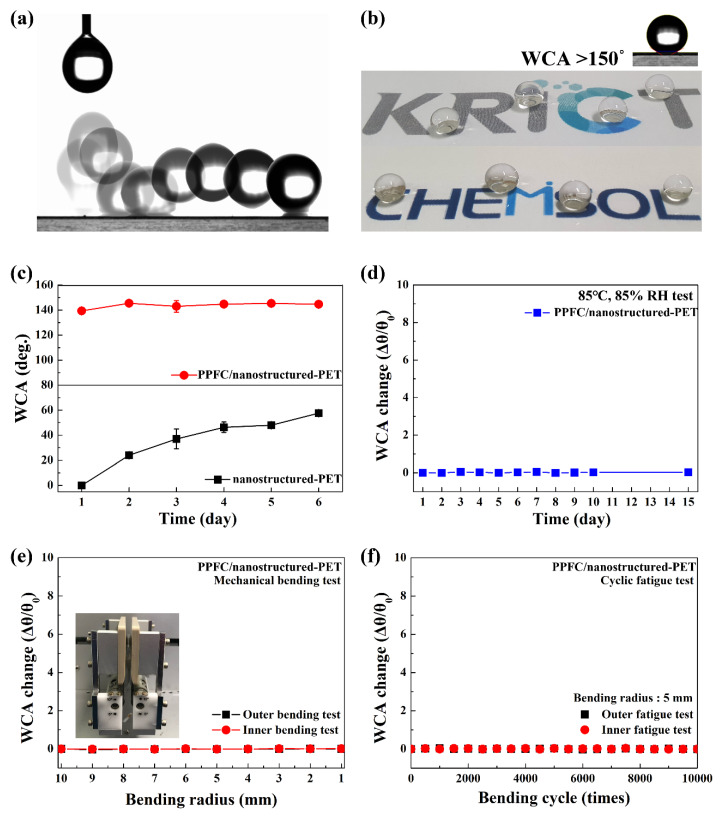
The superhydrophobic and durable characteristics of PPFC/nanostructured-PET: (**a**) Image obtained from a video of water droplets falling on the surface, (**b**) image of water droplets on the PPFC/nanostructured-PET surface, (**c**) aging effect and anti-aging effect results obtained by measuring the WCA change over time, and (**d**) accelerated life test, (**e**) mechanical bending test, and (**f**) cyclic fatigue test of PPFC/nanostructured-PET.

The surface of nanostructured-PET was superhydrophilic due to the high surface energy caused by the oxygen plasma treatment. By coating PPFC with a low surface energy on nanostructured-PET with a superhydrophilic surface, a superhydrophobic surface was realized. Figure 5a shows an image obtained from the video (Video S1 of Supplementary Materials) of a phenomenon in which, when water is dropped on the PPFC/nanostructured-PET surface, it does not adhere to the surface due to its superhydrophobicity and bounces off. In Figure 5b and Supplementary Video S2, the WCA of more than 150° for stationary water droplets shows that the PPFC/nanostructured-PET surface is superhydrophobic. The superhydrophobicity of nanostructured surfaces is the Cassie–Baxter theory which models a heterogeneous surface as if water droplets are in an air pocket [30,31]. The apparent contact angle (*θ*_c_) of the uniformly structured surface is described by the equation cos*θ*_c_ = *f*_1_cos*θ*_1_ − *f*_2_ where *f*_1_ and *f*_2_ are the area fractions of solid–liquid and liquid–air. The *f*_1_ and *f*_2_ of the PPFC/nanostructured-PET surface obtained by the AFM analysis are 12% and 88%, respectively. The WCA calculated using them is 156°, which is highly consistent with the experimental values.

The nanostructures formed on the polymer substrate using the oxygen plasma may exhibit an aging phenomenon in which the structure is restored to its original shape over time. It is known that the aging phenomenon is due to an increase in the amount of oxygen-containing functional groups on the surface and their diffusion into the bulk. Consequently, the surface energy decreases because the polarity on the surface gradually diminishes [32]. In addition, the diffusion and reaction of free radicals and the recontamination of plasma-treated surfaces by contaminants present in the air can also affect aging phenomena [1]. The bottom graph in Figure 5c shows the WCA change over time for the nanostructured-PET surface. The superhydrophilic surface retained its characteristics for one day. However, the WCA rapidly increased from the second day, and, after 6 days, it tended to saturate to approximately 60°. The top graph in Figure 5c shows the WCA change over time for a PPFC/nanostructured-PET sample. Due to the coating of PPFC on nanostructured-PET, the WCA remains the same after a long time. This is because the surface energy of the C–F functional group in PPFC is low and the C–F bond is very stable so that the reaction with the external environment does not occur [19]. Therefore, it can be confirmed that the PPFC coating has an excellent ability to maintain the nanostructure by preventing the aging phenomenon. Eventually, it becomes possible to continuously maintain the superhydrophobic property.

In addition to anti-aging, improving the durability and reliability of superhydrophobic surfaces is a very important issue that needs to be resolved [33,34]. Figure 5d shows the changes in the WCA of the PPFC/nanostructured-PET surface for 15 days in an environment with a temperature of 85 °C and a relative humidity of 85%. It shows that, initially, the WCA was stably maintained without any change even in the high-temperature and high-humidity environment. This result indicates that PPFC/nanostructured-PET has chemical and structural stability even after prolonged environmental exposure. Figure 5e shows the results of the bending test that confirm the mechanical reliability of PPFC/nanostructured-PET. During the bending test, consistent results were obtained even at the bending radius of 1 mm for the inner and outer bending of the film without changing the WCA, which indicates that PPFC/nanostructured-PET has a high mechanical flexibility. Figure 5f shows the results of the cyclic fatigue test obtained by measuring the change in WCA after 10,000 bending cycles at a bending radius of 5 mm at 100 mm/s to evaluate the long-term reliability of mechanical flexibility. For 10,000 bending cycles, the WCA maintained a very constant value above 150°. This indicates that PPFC/nanostructured-PET has a very high mechanical flexibility and excellent durability and maintains superhydrophobic properties even in harsh environments. In conclusion, PPFC/nanostructured-PET, which has an optically high transmittance, excellent antireflective property, and a superhydrophobic surface, has significant applications in flexible displays, large-area solar cells, automotive, and architectural functional film industries, among others.

## 4. Conclusions

In this study, an oxygen plasma was used to form nanostructures, which consisted of nanopores with a diameter of approximately 200 nm and nanoprotrusions with a height of 400 nm on the PET surface using LIS equipment. Nanostructured-PET has a superhydrophilic surface because of its high surface energy due to the presence of oxygen-containing functional groups generated by plasma deformation. Due to the coating of a 100-nm-thick PPFC thin film on the nanostructured-PET surface by MF sputtering, excellent optical properties were obtained (a 91.8% transmittance and a 4.2% reflectance). Particularly, due to the antireflective effect, the reflectance of PPFC/nanostructured-PET exhibited a 54% reduction as compared to the pristine PET substrate. This was due to the combination of the gradient refractive index effect due to the nanostructure and the effect of PPFC coating with a low refractive index. The increase in transmittance due to the PPFC coating also led to a considerable haze reduction.

In addition, due to the low surface energy of PPFC, the superhydrophilic nanosurface changed to a superhydrophobic surface with a WCA of 156°. Therefore, it is possible to fabricate a PET substrate with antireflective as well as self-cleaning properties due to a superhydrophobic surface. The nanostructures formed by the oxygen plasma treatment on the polymer PET surface showed an aging phenomenon over time. This aging phenomenon could be prevented by the PPFC coating, and the surface did not change with time, which exhibits excellent durability characteristics to maintain superhydrophobicity in a high-temperature and high-humidity environment. In addition, the mechanical flexibility for bending and the lifetime of PPFC/nanostructured-PET were evaluated. The superhydrophobic surface was maintained even at the outer and inner bending radius of 1 mm. Moreover, even after 10,000 bending cycles at 100 mm/s at a bending radius of 5 mm, the WCA was consistently maintained at above 150°. In conclusion, nanostructures were uniformly fabricated on the PET surface using an oxygen plasma without a mask. A PPFC thin film was coated on the nanostructured-PET substrate by sputtering to produce an antireflective and superhydrophobic film with excellent durability and lifetime. The antireflective and superhydrophobic films fabricated by the oxygen plasma treatment without a pattern mask and sputtering using polymer composite targets are expected to be quickly applied in the industry since they are capable of large-area processing. Moreover, they can be used in various applications such as in flexible displays, large-area solar cells, energy-saving vehicles, and architecture. 

## Supplementary Materials

The following are available online at https://www.mdpi.com/2073-4360/12/5/1026/s1. Figure S1: the effects of (a) power and (b) oxygen flow rate during oxygen plasma treatment. Table S1: the AFM results of the nanostructured-PET. Table S2: the atomic concentration for the untreated-PET film and nanostructured-PET film. Video S1: a video of water droplets falling on the PPFC/nanostructured-PET surface. Video S2: a video showing superhydrophobic and self-cleaning properties for the PPFC/nanostructured-PET film.
